# Effect of calcium hydroxide on morphology and physicochemical properties of *Enterococcus faecalis* biofilm

**DOI:** 10.1038/s41598-022-11780-x

**Published:** 2022-05-09

**Authors:** Mahere Momenijavid, Himen Salimizand, Aazam Korani, Omid Dianat, Bijan Nouri, Rashid Ramazanzadeh, Amjad Ahmadi, Jino Rostamipour, Mohammad Rastegar Khosravi

**Affiliations:** 1 Student Research Committee, Kurdistan University of Medical Sciences, Sanandaj, Iran; 2 Cellular and Molecular Research Center, Research Institute for Health Development, Kurdistan University of Medical Sciences, Sanandaj, Iran; 3 Food Laboratory, Vice Chancellor for Food and Drug, Kurdistan University of Medical Sciences, Sanandaj, Iran; 4 Division of Endodontics, Department of Advanced Oral Sciences and Therapeutics, School of Dentistry, University of Maryland, Baltimore, MD USA; 5 Social Determinants of Health Research Center, Research Institute for Health Development, Kurdistan University of Medical Sciences, Sanandaj, Iran; 6 Department of Epidemiology and Biostatistics, Faculty of Medicine, Kurdistan University of Medical Sciences, Sanandaj, Iran; 7 Department of Microbiology, Faculty of Medicine, Kurdistan University of Medical Sciences, Sanandaj, Iran; 8 Department of Endodontics, Faculty of Dentistry, Kurdistan University of Medical Sciences, Sanandaj, Iran

**Keywords:** Microbiology, Antimicrobials, Biofilms

## Abstract

Calcium hydroxide Ca(OH)_2_ has been used as an intracanal medicament to targets microbial biofilms and avert secondary infection in the root canal system. This study evaluated the effects of this material on the morphology and physicochemical properties of an established *in-vitro* biofilm of *Enterococcus faecalis*. A biofilm of *E. faecalis* was grown in multichannel plates. The chemicals including Ca^2+^, OH^−^, and saturated Ca(OH)_2_ (ie 21.6 mM) were prepared in order to evaluate which component eradicated or amplified biofilm structure. Various biochemical and microscopic methods were used to investigate the properties of the biofilm. Biofilms treated with Ca(OH)_2_ absorbed more Ca^2+^ because of the alkaline pH of the environment and the ions affected the physicochemical properties of the *E. faecalis* biofilm. A denser biofilm with more cavities and a granular surface was observed in the presence of Ca^2+^ ions. This resulted in a decrease in the surface-to-biofilm ratio with increases in its biomass, thickness, colony size, and volume. Calcium hydroxide did not destroy *E. faecalis* biofilms but rather contributed to the biofilm structure. This *in-vitro* study sheds light on a missing link in the formation of *E. faecalis* biofilm in which the Ca^2+^ in Ca(OH)_2_

## Introduction

Apical periodontitis is a biofilm-induced inflammatory response around the apex of a tooth, which is caused by microbial infections of the root canal system^1^. Prospective biofilm complications for periapical lesions and tissue breakdown can lead to an inflammation that critically necessitates endodontic treatment^2^. The anatomical complexity of the root canal system and organization of microorganisms into biofilms are major factors contributing to the difficulty of elimination of root canal infections^3,4^.

A biofilm is a surface-attached microbial community encased in a self-produced slimy matrix or extracellular polymeric substance (EPS), that comprises 95–98% EPS and 1–5% microorganisms^5^. EPS is primarily composed of water (approximately 90%), polymeric substances (such as alginate and polysaccharides), proteins, eDNA, and lipids^6^. The structural properties of biofilm makes it more resistant to antimicrobial agents and environmental stresses compared to planktonic cells^7^.

Divalent cations such as calcium and magnesium have been shown to cross-link with the polymer strands to provide more stable attachment of the biofilm^8^. The negative charge of EPS is important during its interaction with polyvalent cations. Calcium can induce production of extracellular material such as alginate in mucoid bacterial strains^9^. It has also been suggested that calcium, which binds to cell walls, may play a key role in cell-to-cell adhesion in biofilms as well as stabilization of bacterial cell walls^10^.

Calcium hydroxide Ca(OH)_2_ is widely used as a temporary root canal medicament to hinder the regrowth of bacteria in the root canal between treatment sessions^11^. One of the most important reasons to use it in endodontics is its antimicrobial property. The antimicrobial activity of Ca(OH)_2_ relies on the release of hydroxyl ions (OH^-^) in the presence of water, which results in cytoplasmic membrane distortion, protein denaturation, and DNA damage^12^. However, some studies have reported that calcium hydroxide has limited effectiveness in eliminating bacteria from biofilm^5,13^.

In 2012, van der Waal and van der Sluis hypothesized that calcium plays a key role in the formation of the scaffold and extracellular matrix of a biofilm^14^. They observed that exposure to calcium resulted in distinct changes in the biofilm. They suggested that chelators like Ethylenediaminetetraacetic acid (EDTA) can absorb these calcium ions in the EPS and facilitating the disruption of biofilm. With this as a basis, we investigated the effect of calcium hydroxide ingredients (Ca^2+^ and OH^-^) on the morphology and physicochemical properties of *E. faecalis* biofilms. This bacterium has been commonly used in endodontic biofilm models because it has been coupled with endodontic treatment failures^13,15^.

The antimicrobial properties of this molecule against planktonic microbial cells have been described but its action against biofilm results to be unclear and controversial. The null hypothesis of this study was that calcium hydroxide does not show antibiofilm activity but rather contributes to the biofilm matrix architecture. 

## Results

This laboratory study investigated the effect of calcium hydroxide, calcium and hydroxyl ions on 21-day-old *E. faecalis* biofilms, grown on different artificial surfaces. Evaluation included microscopy (SEM, CLSM, LM, AFM) and determination of calcium and polysaccharide content of the biofilm as well as quantity of viable cells. It was found that the different chemical formulations affected the biofilm morphology and physicochemical properties.

### Biofilm surface morphology

**Figure 1:**
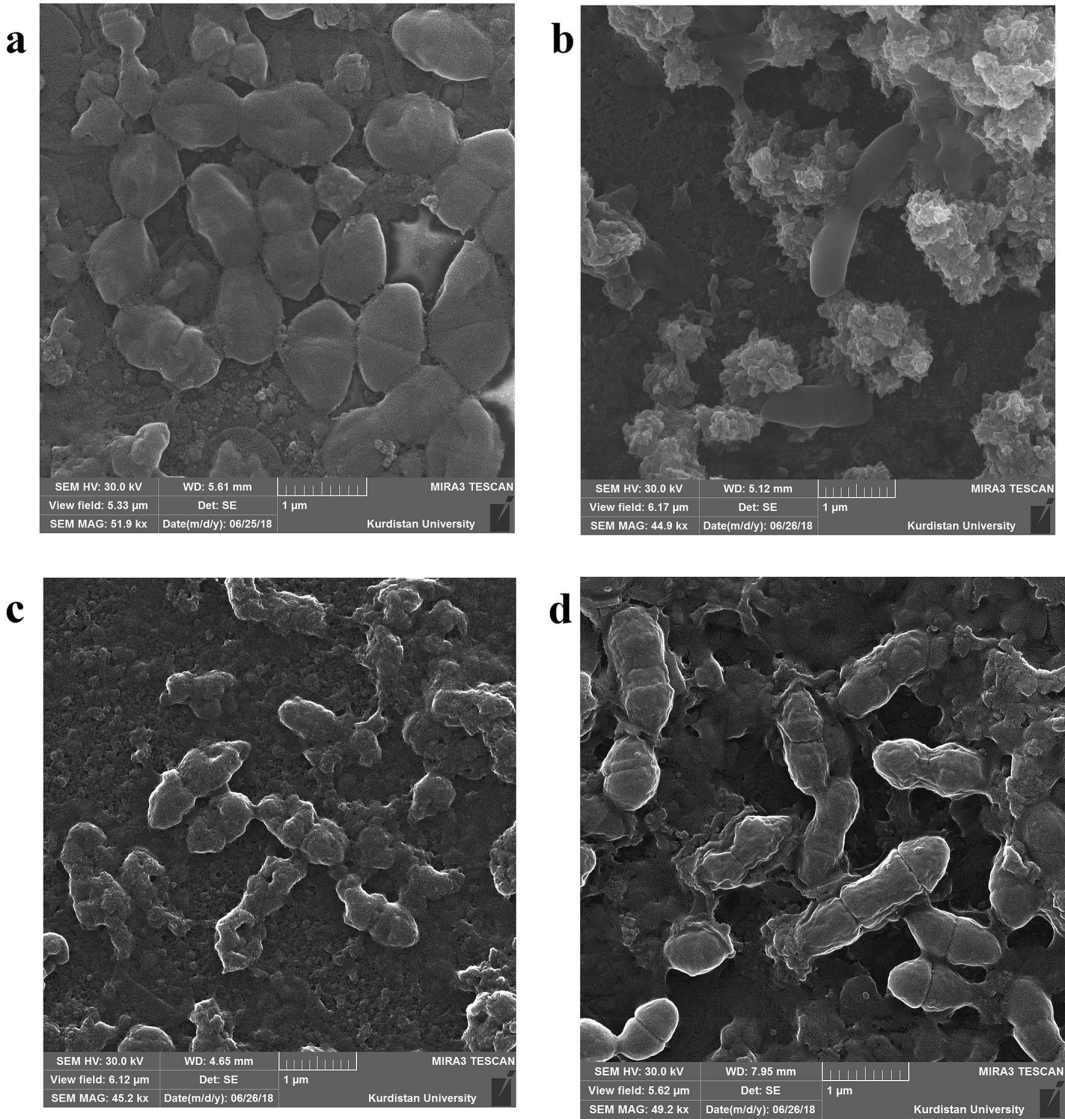
SEM images of *E. faecalis* mature biofilm treated with (**a**), Ca(OH)_2_; (**b**), Ca^2+^; (**c**), OH^-^ for 7 days. The control group, d, was original biofilm without any treatment. The scale bar is 1 µm.

Microscopy showed differences of biofilm growth under different solutions. Scanning electron microscopy (SEM) images revealed that in the Ca(OH)_2_ group an increase in cell size was observed (Fig. 1b). Furthermore, the cells had changed from oval to cigarette-shaped and a small number of them were dividing. Empty holes and a large volume of EPS also was observed in this group. In the Ca^2+^ group, the cell sizes were normal and some of them were dividing. In the OH^-^ group, an increase in cell size was observed. These cells also had a smooth surface.

In the Ca(OH)_2_ and Ca^2+^ groups, biofilm surface was granular due to the huge amount of EPS while in the control and OH^-^ groups, it was smooth (Fig. 1). The granular surface could have been the result of the Ca^2+^ ion bonds, which produced different morphologies when compared with other groups. Furthermore, the SEM images (Fig. 1) showed that in the Ca(OH)_2_ group, the biofilm density was increased, while, in the OH^-^ group, no further biofilm was formed. These observations confirmed the role of Ca^2+^ ions in biofilm progression.

### 3D structure of biofilm

**Figure 2:**
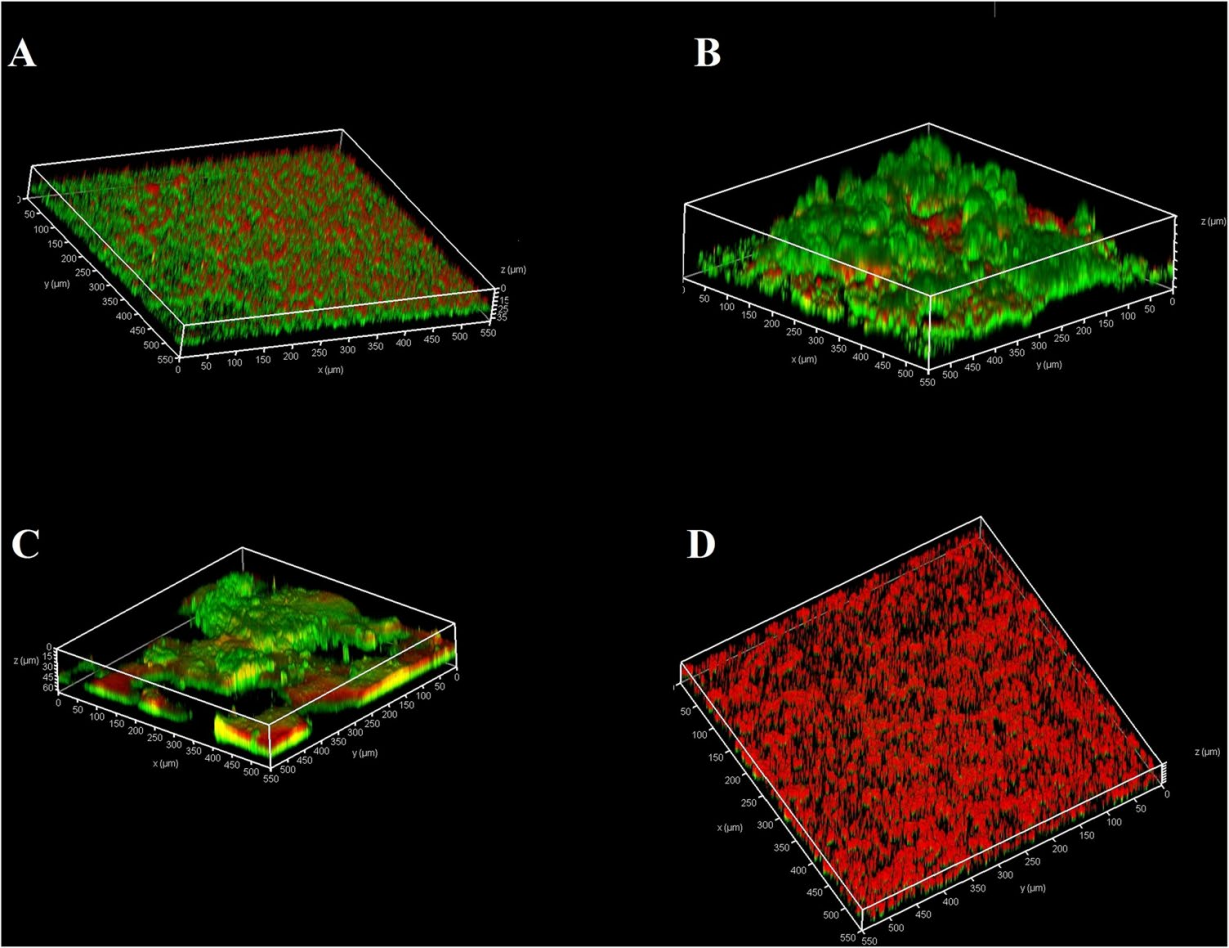
CLSM images of 21-day biofilm of *E. faecalis* that had been treated with solutions for 7 days. The control group was not treated with chemicals. (**A**), control group; (**B**), Ca(OH)_2_; (**C**), Ca^2+^; D, OH^-^.

According to the confocal laser scanning microscopy (CLSM) data, the structural properties of biofilm was described in terms of thickness, colony volume, biomass, colony size and surface-biovolume ratio (Supplementary Table S1 and S2). The CLSM images showed that the presence of Ca^2+^ ions induced structural changes and a denser biofilm formation (Fig. 2). In the control group, the biofilm covered a larger surface, but its volume and the thickness were smaller and contained living cells (as expected).

**Figure 3:**
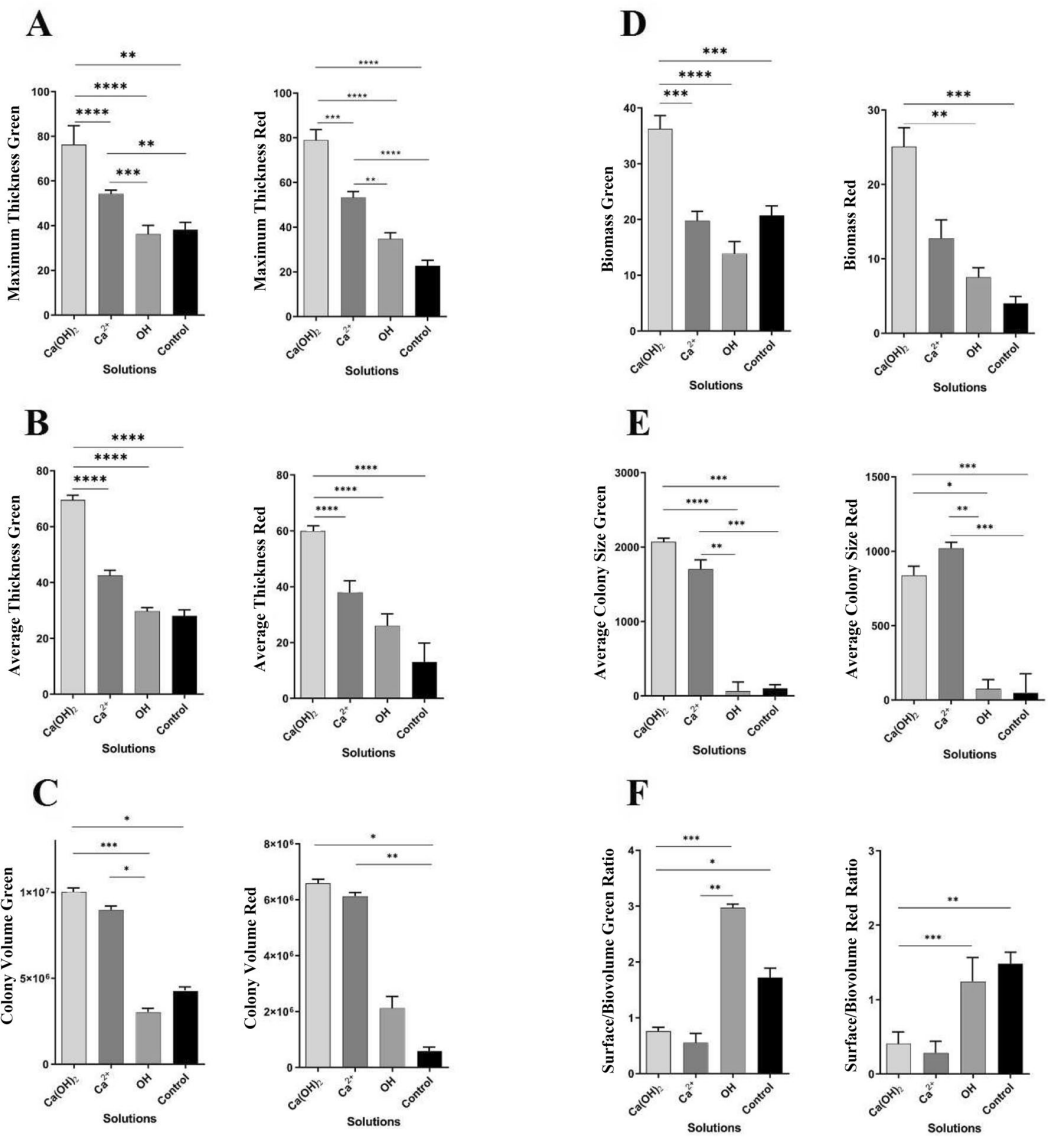
Calculated amount of CLSM parameters and related *P* value. (**A**) Maximum thickness; (**B**) Average thickness; (**C**) Colony volume; (**D**) Biomass; (**E**), Average colony size; (**F**), Surface to biomass ratio. Significant token: *, 0.05; **, 0.01; ***, 0.001; ****, 0.0001. No significant results were not illustrated.

The maximum thickness of the green and red dyed biofilm was considerably different between groups. The Ca(OH)_2_ group had the most biofilm thickness followed by Ca^2+^ group. The OH^-^ and control groups had the least biofilm thicknesses (Fig. 3A, Tables S1 & S2).

The Ca(OH)_2_ group had significantly highest average biofilm thickness (*p*-value < 0.0001) followed by the Ca^2+^, OH^-^, and control group (Fig. 3B, Tables S1 & S2). Statistical analysis indicated that the average colony volume related to the green and red dyes were significant. The Ca(OH)_2_ group was significantly different (p-value < 0.0001) from other groups (Fig. 3C, Tables S1 & S2). This group formed significantly much more biomass than did the other groups (*p*-value < 0.0001). The difference between the other groups was not significant (Fig. 3D, Tables S1 & S2). The Ca(OH)_2_ and Ca^2+^ groups had larger colony size; meanwhile, the OH^-^ and control groups formed smaller one (Fig. 3E, Tables S1 & S2). The OH^-^ group had the most surface/volume ratio followed by the control, Ca(OH)_2_, and Ca^2+^ groups (Fig. 3F, Tables S1 & S2).

### Morphology and roughness of the bacterial biofilm

**Figure 4:**
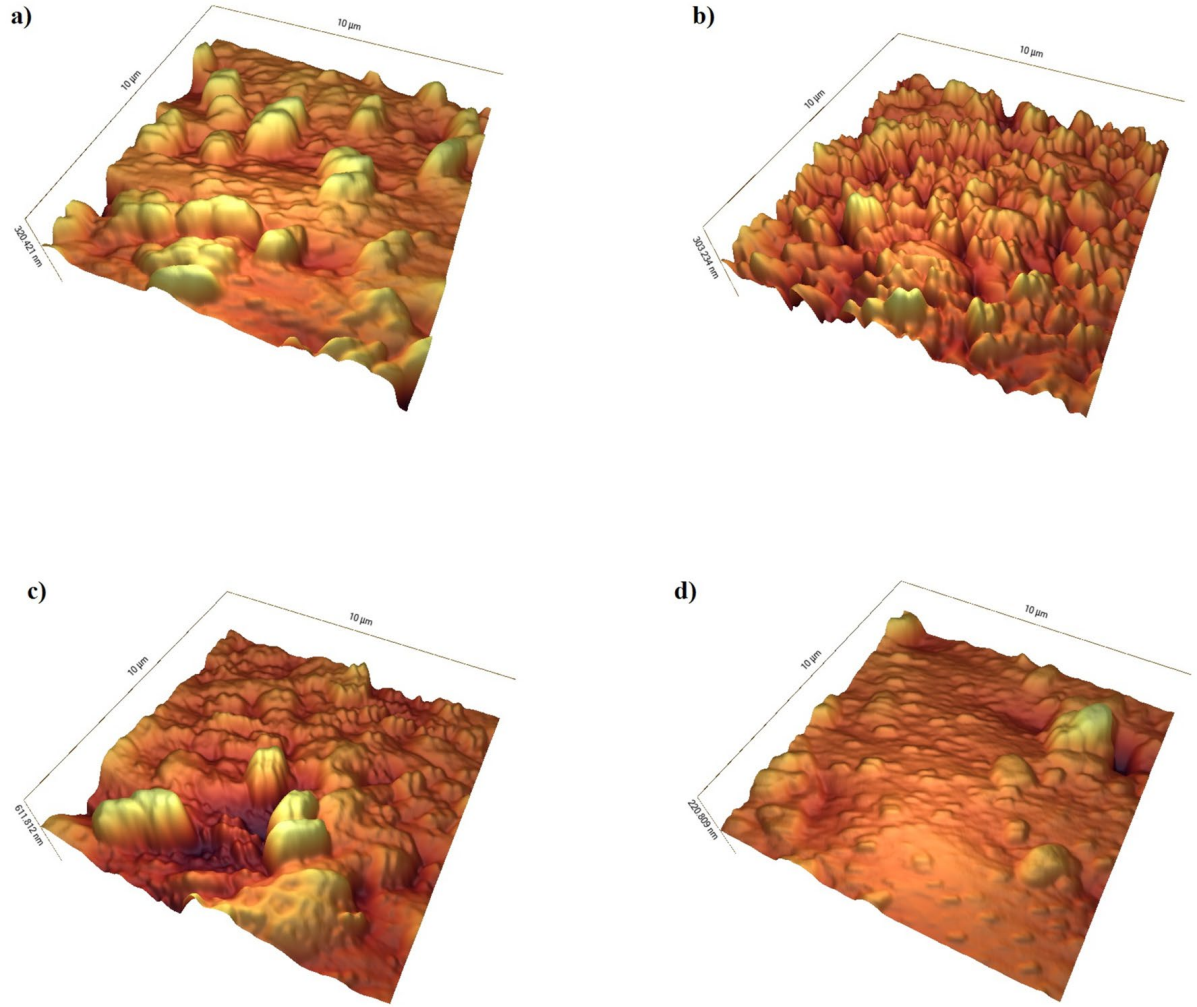
AFM images of a 21-day biofilm of *E. faecalis* after 7 days under different solutions, at a scale of 10 µm^2^. (**a**) control (43.4); (**b**) Ca(OH)_2_ (52.6); (**c**) Ca^2+^ (56.7); (**d**) OH^-^(42).

The atomic force microscopy (AFM) microscopy images revealed the variable morphology and roughness of the bacterial biofilms (Fig. 4). The roughness values of the samples were 52.6, 56.7, 42, and 43.4 for the Ca(OH)_2_, Ca^2+^, OH^-^, and control groups, respectively. Statistical analysis using the Kruskal–Wallis test showed no significant difference between Ca(OH)_2_ and Ca^2+^groups (*P* = 0.727). Meanwhile, the morphological difference pertinent to Ca(OH)_2_ and OH^-^ groups revealed the cooperation of calcium in biofilm progression where the environment was alkaline.

#### Polysaccharide staining of biofilm

**Figure 5:**
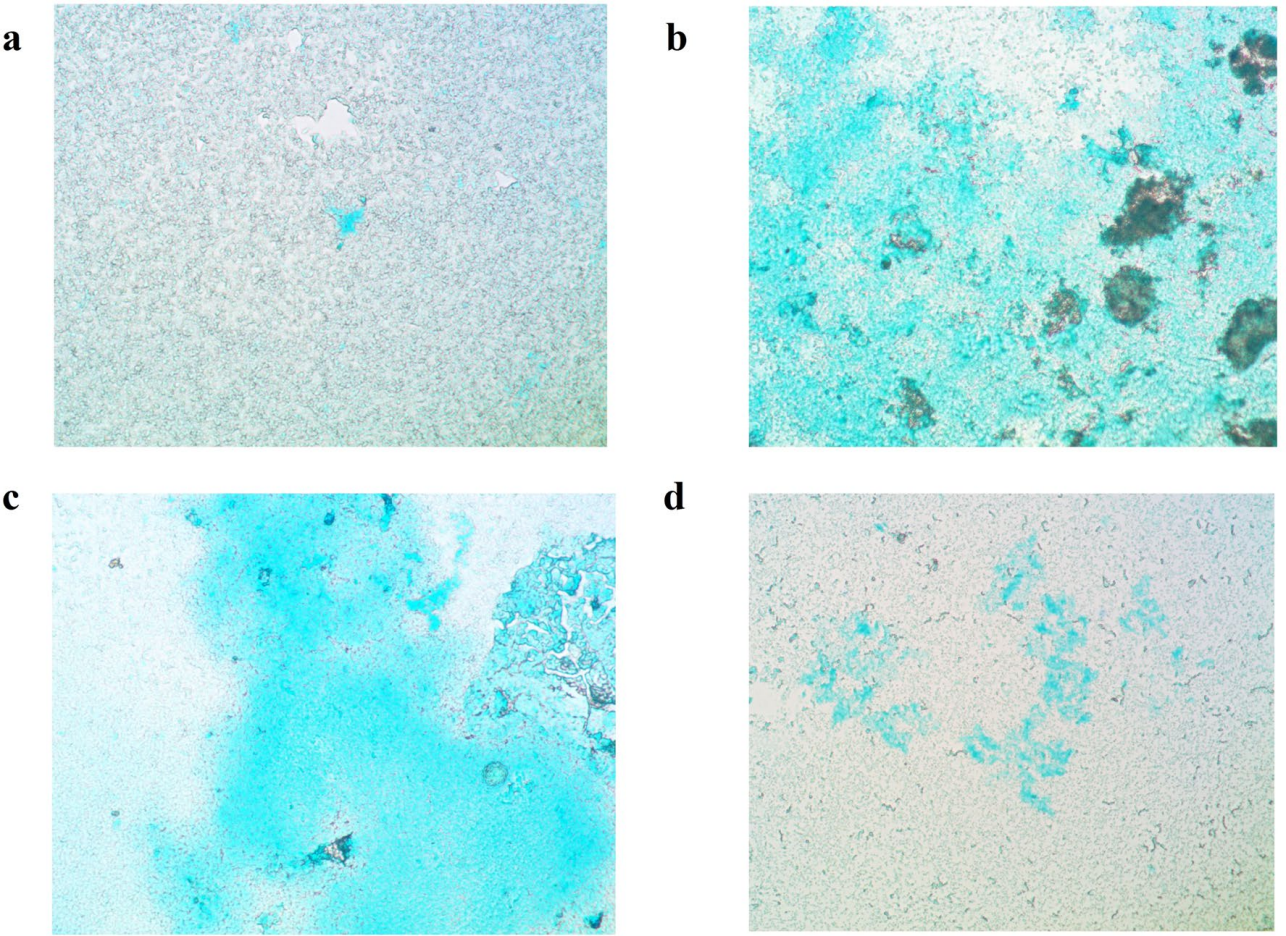
Light microscopy images of stained 21-day biofilm polysaccharides of *E. faecalis* after 7 days of contact with solutions. (**a**), Control, no solution; (**b**), Ca(OH)_2_; (**c**), Ca^2+^; (**d**), OH^-^.

The amount of total polysaccharide increased in the presence of free Ca^2+^ ions in the Ca(OH)_2_ (Fig. 5b) and Ca^2+^ (Fig. 5c) groups. However, the amount of polysaccharide that formed in the OH^-^ group (Fig. 5d) was very low and similar to that of the control group (Fig. 5a).

### Quantification of biofilm polysaccharide

**Figure 6:**
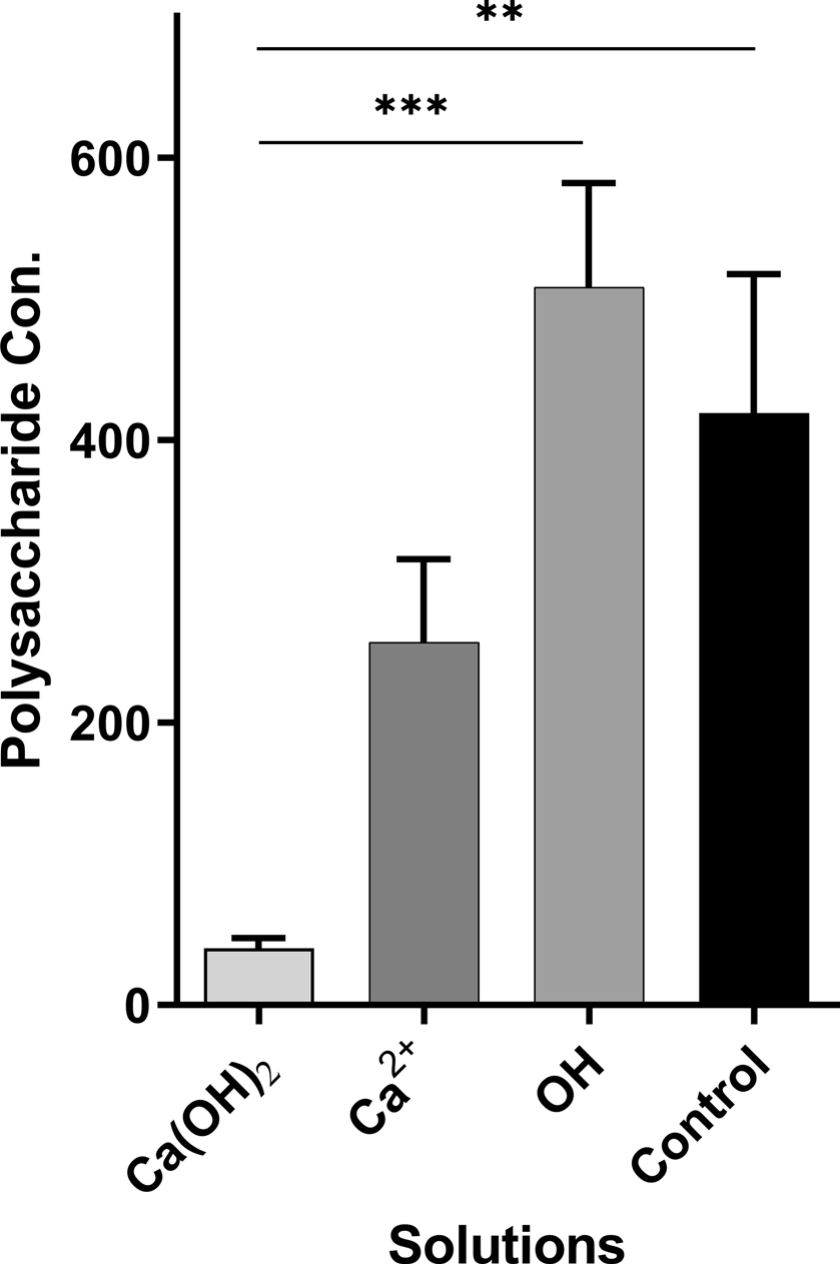
Measured polysaccharide concentrations by groups. Significance token: *, 0.05; **, 0.01; ***, 0.001; Non-significant results are not shown.

The mean concentration of free polysaccharides was measured for in all groups (Fig. 6). A remarkable difference was observed between groups. Overall, it could be concluded that the concentration of polysaccharides in the Ca(OH)_2_ group was significantly different from the other groups.

### Calcium measurement of biofilm

**Figure 7:**
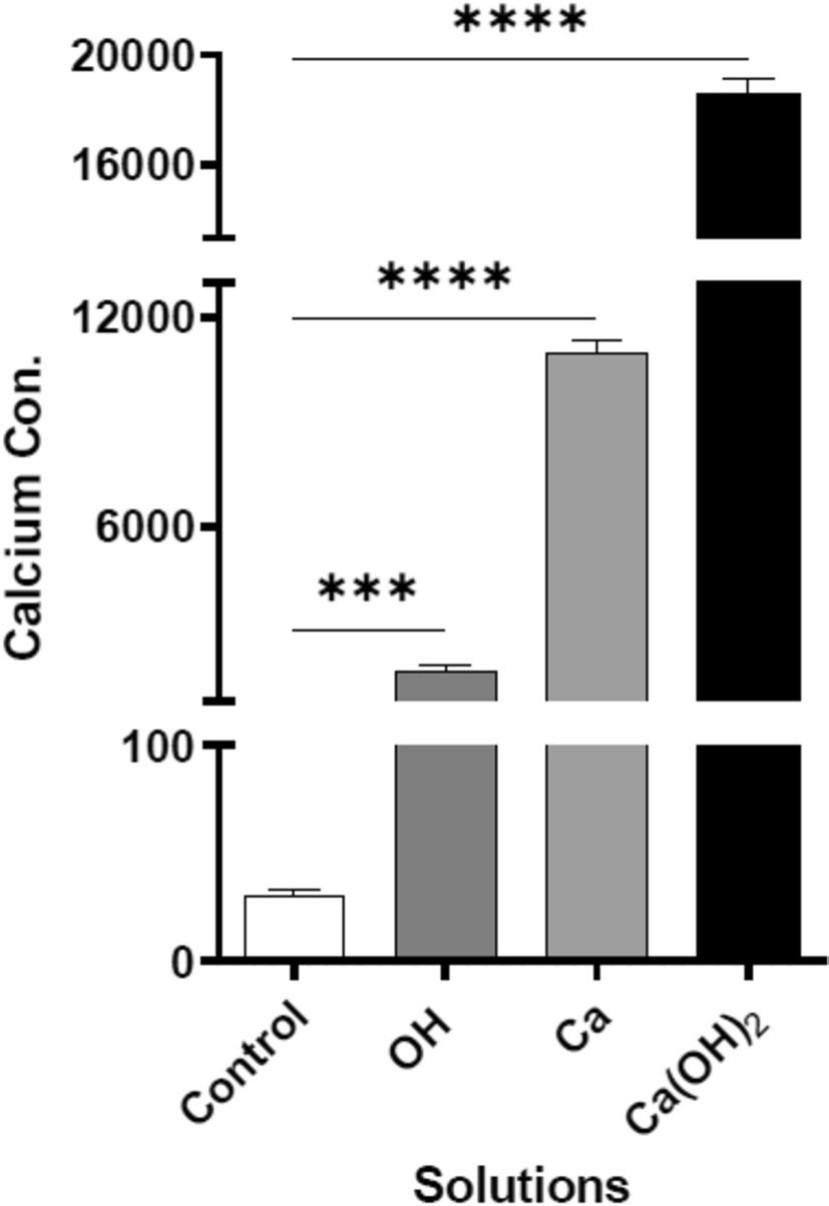
Ca^2+^ concentration of biofilm in different groups (*P* < 0.05). Significance: *, 0.05; **, 0.01; ***, 0.001; ****, 0.0001. Non-significant results are not shown.

The Ca^2+^ ions content of the biofilm was measured by ICP technique. The results showed that the mean Ca^2+^ concentration in the control, Ca(OH)_2_, Ca^2+^, and OH^-^ groups were 30.8, 18,555, 10,948 and 2211 ppm, respectively (Fig. 7). The Ca^2+^ ions content of the biofilm was at maximum levels in the Ca(OH)_2_ group, which was in compliance with previous results and confirmed that Ca^2+^ ions played major role in biofilm progression in alkaline environment.

### Quantity of viable cells in biofilm

**Figure 8:**
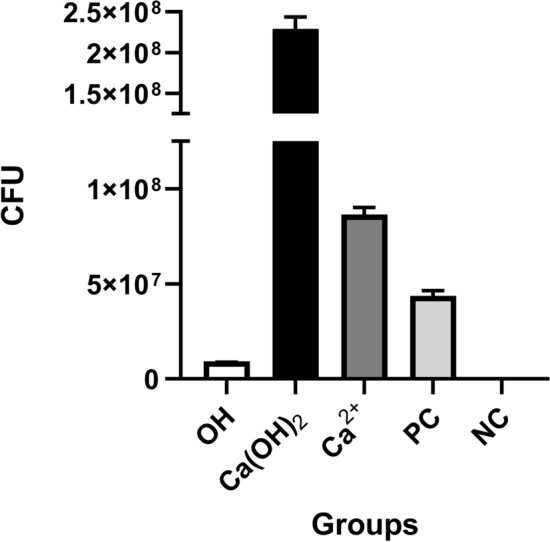
Quantitation of viable cells under different solutions. Abbreviations: Negative control, NC; Positive control, PC.

The number of surviving bacteria in the OH^-^ group was lowest followed by the Ca^+2^, Ca(OH)_2_, and control groups (Fig. 8). This demonstrates the protective nature of the biofilm under a higher concentration of Ca^+2^, although the alkaline environment disrupted the viable cells.

## Discussion

This study investigated the effect of calcium hydroxide on the morphology and physiochemical properties of *E. faecalis* biofilms. SEM, CLSM, and AFM methods were used to evaluate the morphologic and structural properties; whereas, viable cells in the biofilm, polysaccharides, Ca^2+^, and EPS components were measured to assess the chemical properties of the biofilms. The results revealed that Ca(OH)_2_ components, ie Ca^2+^, and alkaline pH, through a cooperative manner strengthen biofilm. Therefore, Ca(OH)_2_ did not eradicate *E. faecalis* biofilm, but promoting growth of biofilm.

The EPS of the biofilm in the Ca(OH)_2_ and Ca^2+^ groups formed a granular surface; while, in the control and OH^–^ groups, the surface was smooth. The granular surface was produced by the Ca^2+^ ion bonds, which led to morphological differences when compared to the smooth surfaces in the absence of Ca^2+^ ions. These findings are in consistent with those of Safari et al*.*,who showed that the addition of Ca^2+^ to the biofilm produced a granular surface^16^. Moreover, Mangwani et al*.* observed that the addition of Ca^2+^ caused cavities in the biofilm. An increased in cavities in the biofilm indicates the accumulation of large amounts of EPS^17^.

In regard to the qPCR and CLSM results, it can be concluded that most of the green dye displayed in the Ca(OH)_2_ and Ca^2+^ groups were live cells, while in the OH^-^ group the number of viable cell dramatically reduced. In the Ca(OH)_2_ group, because of alkaline pH of the environment, the uptake of Ca^2+^ increased in the biofilm such that number of living bacteria was more than other groups^18^. This finding is consistent with the report pertinent to elevated biomass by adding Ca^2+^ to the biofilm^9,16,17,19^.

The results of quantitative analysis of free polysaccharides in the EPS revealed that the lowest concentration of free polysaccharides was for the Ca(OH)_2_ group. This can be explained by the mechanisms of the Smith–Gilkerson reaction, in which the OH part of the sixth carbon of the saccharide ring in the polysaccharide structure, used to produce chromogenic substance, was associated with large amounts of Ca^2+^ ions and was unable to interact with the Smith–Gilkerson reagent. We concluded that most of the polysaccharides in the biofilm were not free and had not been measured by the Smith–Gilkerson method.

In conclusion, the presence of Ca^2+^ ions caused a denser biofilm with more cavities and indicates an increase in EPS. The presence of this ion also created a granular surface in the biofilm. Expansion of the biomass and increases in the thickness, colony size, and volume of the biofilm as well as declining the surface-to-biofilm ratio were the results for the Ca^2+^ ions in the biofilm. The alkaline pH of the environment enhanced absorption of Ca^2+^ when a biofilm was treated with Ca(OH)_2_; thus, this ion was able to affect the morphology, structure, and chemical properties of the *E. faecalis* biofilm as well.

This study has shown that Ca^2+^, as a part of calcium hydroxide, serves a missing link in the biofilm progression of *E. faecalis*. Calcium hydroxide does not destroy the biofilm of *E. faecalis*, but actually participates in strengthening the biofilm of this bacterium.

## Material and methods

### Solution preparation and biofilm assay

In this study, the standard *E. faecalis* strain (ATCC 29,212) was used in all experiments. Brain heart infusion (BHI) agar (Merck; Germany) and BHI broth culture medium (Merck; Germany) were prepared according to manufacturer instructions. The isolated colonies were cultured on BHI agar plates and incubated under aerobic conditions at 37 °C for 24 h. Thereafter, a bacterial suspension in normal saline was prepared adjusted to the 0.5 McFarland standard and diluted to equal 10^6^ CFU/mL. Chamber slides, cover glass, mica, and falcon tubes were used in which form the biofilm for microscopic observations. To produce mature biofilms, the BHI broth was replaced every 48 h for 21 days. The samples were incubated at 37 °C in ambient air^20,21^.

To prepare the Ca(OH)_2_ solution, 1.65 g of powder was dissolved in 1000 mL of deionized water (i.e. 21.6 mM) and stirred for 3 h. After overnight incubation at room temperature, the solution was centrifuged at 8000 RPM for 10 min. The supernatant was used as an 100% Ca(OH)_2_ solution. To prepare pure Ca^2+^ ions, a part of aforementioned solution was neutralized using glacial acetic acid at pH 7. To produce the OH^–^ ions, 100% Ca(OH)_2_ solution was chelated using ethylenediaminetetraacetic acid (EDTA). Thereafter, the Ca^2+^ content was quantified using Eriochrome Black T indicator solution (Sigma-Aldrich; Germany) to ensure all of the Ca^2+^ had chelated (https://chem.libretexts.org/@go/page/75718). For convenience, the groups were denoted as Ca(OH)_2_, Ca^2+^, and OH^-^.

After 21 days of incubation and formation of a mature biofilm, it was exposed to one of three solutions for 7 days to simulate clinical conditions. All experiments were carried out in triplicate.

### Scanning electron microscopy (SEM)

To assess the surface morphology and composition of the *E. faecalis* biofilm under the different solutions, they were examined with scanning electron microscopy (SEM). For this purpose, a 1 cm × 1 cm cover glass was embedded in 6-well plates (Guangzhou Jet Bio-Filtration; China), and 5 mL of freshly inoculated *E. faecalis* BHI media was poured into each well.

The cumulative number of inoculated bacteria was adjusted to 10^6^ CFU/mL. The biofilm culturing and treatments were the same as for the biofilm assay. Thereafter, the cover glass was placed into 4% glutaraldehyde (Merck; Germany) to fixate the biofilms. After 1 h, the samples were washed with double-distilled water (DDW) and air dried. They then were mounted on a gold-coated SEM stub and photographed using a MIRA3 scanning electron microscope (Tescan; Czech)^22^. The accumulation of bacteria and biofilm formation under different conditions were investigated.

### Confocal laser scanning microscopy (CLSM)

CLSM was used to examine the reconstruction of three-dimensional structures in the biofilm. The biofilm was grown on a chamber slides (Nunc Lab-Tek; Thermo Fisher Scientific; Denmark) and exposed to the solutions as described in the biofilm assay. The slides were stained with fluorescein diacetate (FDA) green fluorescent dye (Sigma-Aldrich; Germany) for live bacteria and with propidium iodide (PI) red fluorescent dye (Sigma-Aldrich; Germany) for dead bacteria. The PI was not able to cross the membrane of living cells but can stain the DNA of dead bacteria or eDNA in EPS.

After staining, the samples were incubated at room temperature for 20 min in a dark place. Then they were gently rinsed with phosphate buffer saline (PBS) to remove non-adherent bacterial cells. For imaging, an inverted microscope (Leica TCS-SPE system; USA) was used at × 10 magnification. The excitation wavelength used for the FDA and PI were 488 nm and 532 nm, respectively, and the emission wavelengths were 500–550 and 580–700 nm, respectively. Four to six regions were randomly selected for each biofilm in each group. The biofilm parameters of biomass, mean thickness, maximum thickness, average colony size, average colony volume, and surface-to-volume ratio were evaluated. The three-dimensional images were analysed using Comstat software (V. 2.1, www.comstat.dk)^17,21,23^.

### Atomic force microscopy (AFM)

The roughness value of *E. faecalis* biofilm surface under the solutions were assessed using AFM. After the biofilm was grown on mica and then put into contact with the solutions, each was washed twice with DDW and dried in a desiccator overnight. The imaging of the prepared samples was done with a JPK NanoWizard II BioAFM (JPK Instruments, Germany) that was combined with an inverted optical microscope (Nikon, Japan) and a Hamamatsu CCD camera, in tapping mode (Mikromasch NSC15 tips) with a scanning speed of 1 line/s. Finally, the images were analysed with JPK IP (Version 3.3, JPK Instruments)^16^.

### Light microscopy

To ensure that changes in the parameters measured by Comstat in the CLSM experiment were not solely due to the changes in the number of bacteria, the biofilms was stained with Alcian blue (Sigma-Aldrich; Germany). The matrix and acidic extracellular polysaccharides of the biofilm became visible. The biofilm was examined with a light microscope (PH2-RFCA Olympus, Japan) at a × 400 magnification^6^.

### Biofilm polysaccharide measurement

The amount of free polysaccharides in the biofilms was quantified using the Smith–Gilkerson method^24^. Biofilm was grown in a 50 mL falcon tube and brought into contact with the solutions, as described previously. The samples were dried in an incubator (3 days; 40 °C), their weight was measured, and the falcon walls were then carefully scraped using a spatula to remove the biofilm. Next, 2.5 mL of DDW was added to each sample. The samples were then sonicated eight times in 30-s cycles at 60% power with the a sonicator (Ultrasound Technology, UP200h; Germany) to release the polymers from the bacterial cells.

These samples then were centrifuged at 5000 g for 30 min at 12 °C to separate the supernatants from the bacterial cells. A volume of 1.5 mL of the clear supernatant was removed from the suspension and centrifuged again for 10 min at 12,000 g. Finally, 1 mL of the final clear supernatant was used to determine the polysaccharides concentration. The polysaccharide compounds were hydrolyzed using 0.5 M of hydrochloric acid (Merck; Germany). Then, 3-methyl-2-benzothiazolinone hydrazine (MBTH) (Sigma-Aldrich; Germany), a chromophore, formed a colour complex and the optical density was measured at 650 nm.

### Biofilm calcium content

Inductively coupled plasma was used to measure the amount of calcium in the biofilm matrices. In four falcon tubes, *E. faecalis* was grown to form biofilms and then was put into contact with the solutions as described previously. The samples were dried in a desiccator and the falcons were weighted. Then, 1 mL of nitric acid (Merck, Germany) and 1 mL of hydrogen peroxide (Merck, Germany) were added to each sample. Finally, the volume of each sample was increased to 10 mL with DDW and Ca^2+^ ion concentration was measured by inductively coupled plasma-optical emission spectroscopy (OES-ICP; 730-ES; Varian; USA) at a wavelength of 397/847 nm^25^.

### Quantification of surviving cells in biofilm

The biofilm was prepared in 96-well flat-bottom plates for each group and for positive (non-treated) and negative (vancomycin-treated) controls. After 21 days of biofilm formation, the plates were gently rinsed twice with PBS to remove the unattached bacteria. To enumerate the viable cells, the RNA of each well was harvested using TRI reagent (Sigma Aldrich; Germany) following manufacturer protocols with a few modifications. Briefly, 100 µL of lysozyme (20 mg/mL) was added to each well for 10 min to reduce the EPS. Then, RNase free DNase (ThermoFisher Scientific) was poured into each well to digest the eDNA. Finally, 100 µL of TRI reagent was used in each well followed by RNA extraction. The upper phase containing RNA was transferred to a RNeasy column extraction kit (Takara Bio; Japan) to prepare the pure RNA. DNase treatment was performed to eliminate probable DNA contamination.

The purity and quantity of RNA were detected using NanoDrop 1000 spectrophotometer (Thermo Scientific, Waltham, MA; USA). The cDNA was constructed using a commercial kit (Takara Bio; Japan). Quantitative real-time polymerase chain (qPCR) reaction was done to determine the absolute number of viable bacteria in the biofilm using SYBR reagent (Takara Bio; Japan) and specific primers^26^ on a C1000 Touch™ Thermal Cycler (Bio-Rad;PA; USA). The RNA content was translated into the bacterial content in accordance with a standard curve and was reported as colony forming units (CFU) per mL. The experiment carried out in triplicate.

### Data analysis

The normality of the continuous variables was assessed using the Shapiro–Wilk test. In order to check the parameters of CLSM images and polysaccharide quantity, one-way ANOVA and Bonferroni post-hoc tests were used, respectively. The roughness values in the AFM and the calcium content in the biofilms were evaluated by applying the Kruskal–Wallis and Mann–Whitney tests. All data analyses were performed using Stata version 12.0 (StataCorp., USA), and *P* < 0.05 indicated statistical significance. The SEM results were descriptively assessed and reported.

## Data availability

The datasets generated during and/or analyzed during the current study are available from the corresponding author on reasonable request.

## Supplementary Information

Supplementary Information.
