# Supplementary material for: Effect of calcium hydroxide on morphology and physicochemical properties of Enterococcus faecalis biofilm
Source: Sci Rep. 2022 May 9;12:7595. doi: 10.1038/s41598-022-11780-x (PMC9085741; doi:10.1038/s41598-022-11780-x)
Supplement: Supplementary file 1 — Supplementary Information. [file 41598_2022_11780_MOESM1_ESM.pdf]

Supplement Table S1. CLSM parameters calculated by Comstat software (V. 2.1)

		$Ca(OH)_2$	Ca^{+2}	OH^-	Control
A. Maximum thickness (µm)	Green	80.499	54.599	36.399	40.559
	Red	80.499	52.499	36.399	23.799
B. Average thickness (µm)	Green	69.288	43.968	29.855	28.252
	Red	59.615	38.094	26.035	13.414
C. Colony Volume (μm^3)	Green	10024788	8960333	3013696	4251200
	Red	6592831	6120697	2116446	588469
D. Biomass	Green	37.991	17.977	13.497	20.058
	Red	24.434	12.073	8.002	3.655
E. Average colony size (μm^3)	Green	2073.787	1703.191	62.715	101.150
	Red	873.893	1019.009	75.024	48.033
F. Surface/Biovolume ratio ($\mu m^2/\mu m^3$)	Green	0.0762	0.554	2.769	1.721
	Red	0.417	0.285	1.240	1.489

Supplement Table S2. P-value of CLSM parameters (significant *p*-values are underlined)

A. Maximum thickness		$Ca(OH)_2$	Ca^{+2}	OH^-
Ca^{+2}	Green	P=0.000		
	Red	P=0.000		
OH^-	Green	P=0.000	P=0.002	
	Red	P=0.000	P=0.015	
Control	Green	P=0.000	P=0.015	P=1.000
	Red	P=0.000	P=0.000	P=0.077
B. Average thickness		$Ca(OH)_2$	Ca^{+2}	OH^-
Ca^{+2}	Green	P=0.000		
	Red	P=0.000		
OH^-	Green	P=0.000	P=0.034	
	Red	P=0.000	P=0.024	
Control	Green	P=0.000	P=0.016	P=1.000
	Red	P=0.000	P=0.000	P=0.017
C. Colony volume		$Ca(OH)_2$	Ca^{+2}	OH^-
Ca^{+2}	Green	P=1.000		
	Red	P=1.000		
OH^-	Green	P=0.007	P=0.026	
	Red	P=0.083	P=0.149	
Control	Green	P=0.032	P=0.109	P=1.000
	Red	P=0.011	P=0.021	P=1.000
D. Biomass		$Ca(OH)_2$	Ca^{+2}	OH^-
Ca^{+2}	Green	P=0.001		
	Red	P=0.093		
OH^-	Green	P=0.000	P=1.000	
	Red	P=0.014	P=1.000	
Control	Green	P=0.002	P=1.000	P=0.647
	Red	P=0.002	P=0.502	P=1.000
E. Average colony size		$Ca(OH)_2$	Ca^{+2}	OH^-
Ca^{+2}	Green	P=1.000		
	Red	P=1.000		
OH^-	Green	P=0.000	P=0.001	
	Red	P=0.045	P=0.015	
Control	Green	P=0.002	P=0.001	P=1.000
	Red	P=0.167	P=0.073	P=1.000
F. Surface/Biovolume ratio		$Ca(OH)_2$	Ca^{+2}	OH^-
Ca^{+2}	Green	P=0.713		
	Red	P=1.000		
OH^-	Green	P=0.000	P=0.000	
	Red	P=0.005	P=0.001	
Control	Green	P=0.000	P=0.000	P=0.000
	Red	P=0.001	P=0.000	P=1.000
